# The Electrostatic Basis of Diacylglycerol Pyrophosphate—Protein Interaction

**DOI:** 10.3390/cells11020290

**Published:** 2022-01-15

**Authors:** Zachary Graber, Desmond Owusu Kwarteng, Shannon M. Lange, Yannis Koukanas, Hady Khalifa, Jean W. Mutambuze, Edgar E. Kooijman

**Affiliations:** 1 Department of Chemistry & Biochemistry, Kent State University, 800 E. Summit St., Kent, OH 44242, USA; 2 School of Natural and Social Sciences, Mount Vernon Nazarene University, 800 Martinsburg Road, Mount Vernon, OH 43050, USA; 3 Department of Biological Sciences, Kent State University, 800 E. Summit St., Kent, OH 44242, USA; shannon.lange@stjude.org (S.M.L.); ykouko01@villanova.edu (Y.K.); hkhalifa@kent.edu (H.K.); jmutambu@kent.edu (J.W.M.)

**Keywords:** protein-lipid interactions, membrane electrostatics, lipid physical chemistry

## Abstract

Diacylglycerol pyrophosphate (DGPP) is an anionic phospholipid formed in plants, yeast, and parasites under multiple stress stimuli. It is synthesized by the phosphorylation action of phosphatidic acid (PA) kinase on phosphatidic acid, a signaling lipid with multifunctional properties. PA functions in the membrane through the interaction of its negatively charged phosphomonoester headgroup with positively charged proteins and ions. DGPP, like PA, can interact electrostatically via the electrostatic-hydrogen bond switch mechanism but differs from PA in its overall charge and shape. The formation of DGPP from PA alters the physicochemical properties as well as the structural dynamics of the membrane. This potentially impacts the molecular and ionic binding of cationic proteins and ions with the DGPP enriched membrane. However, the results of these important interactions in the stress response and in DGPP’s overall intracellular function is unknown. Here, using ^31^P MAS NMR, we analyze the effect of the interaction of low DGPP concentrations in model membranes with the peptides KALP23 and WALP23, which are flanked by positively charged Lysine and neutral Tryptophan residues, respectively. Our results show a significant effect of KALP23 on the charge of DGPP as compared to WALP23. There was, however, no significant effect on the charge of the phosphomonoester of DGPP due to the interaction with positively charged lipids, dioleoyl trimethylammonium propane (DOTAP) and dioleoyl ethyl-phosphatidylcholine (EtPC). Divalent calcium and magnesium cations induce deprotonation of the DGPP headgroup but showed no noticeable differences on DGPP’s charge. Our results lead to a novel model for DGPP—protein interaction.

## 1. Introduction

Anionic signaling lipids such as phosphatidic acid (PA) and the phosphoinositides (PIPs) play crucial roles in the life of a cell [1,2,3]. Their diverse functions are mediated directly by interaction with membrane proteins and/or indirectly by modulation of key membrane properties, such as membrane curvature [4,5,6,7]. Here we focus on the PA metabolite, diacylglycerol pyrophosphate (DGPP), which is formed in plants and parasites upon stress signaling but has not been found in mammalian cells [8,9,10].

PA is present in small (at most a few percent) amounts in biomembranes but increases rapidly in plants after a stress stimulus [11,12]. This is followed by a decline in PA concentration and a subsequent increase in DGPP production as PA is converted to DGPP by phosphatidic acid kinase (PAK) [10]. PAK phosphorylates the phosphomonoester of PA to a pyrophosphate and has been reported in plants such as *Arabidopsis thaliana, Craterostigma plantagineum* and *Vicia sativa* following their treatment with ABA, hyperosmotic stress and Nod factor, respectively [8,13,14]. DGPP formation is purported to not only reduce the effect of PA (levels) but also actively function in the stress response pathway [15]. The signaling function of DGPP may involve either specific interactions with proteins, where protein function is modulated (such as activation and inactivation), or through changes in membrane physical properties such as membrane curvature, bending rigidity, and charge. Additionally, the charge on the pyrophosphate headgroup of DGPP is likely to influence protein–lipid interactions [16].

The ionization properties of DGPP, as elucidated by Strawn et al. in 2012 in model membranes of phosphatidylcholine (PC) and phosphatidylethanolamine (PE), differ from those of PA [17]. The pK_a2_ of the phosphomonoester of DGPP is lower than that of PA due to its location in the lipid headgroup–aqueous interface (with DGPP sitting higher than PA). Additionally, PE lowered the pK_a2_ of this phosphate following the electrostatic-hydrogen bond switch model, which we introduced in 2007 [17,18]. Structurally, the cylindrical shape of DGPP, unlike the cone shape of PA, does not induce a negative membrane curvature (stress) [17]. Differing from PA in both shape and charge, increased DGPP production may alter the dynamics of the membrane. After conversion of PA to DGPP [10], a DGPP enriched biomembrane will have less negative curvature stress and an increased negative surface potential, which is likely to influence the interaction with (cationic) ions and proteins [17]. However, the interaction of DGPP with cationic amino acids and/or divalent cations has not been fully explored and is the main focus of this work [19,20].

Positively charged amino acid side chains, such as Arg, His and Lys, mediate the interaction and anchoring of proteins and peptides to anionic phospholipid headgroups in biomembranes [21,22]. Such electrostatic interactions are vital in signaling pathways and enable the selective targeting of proteins to different lipid domains in biomembranes [23]. NMR and MD simulations on PA and model peptides (KALP and RALP) revealed a hydrogen bond formation between the phosphomonoester of PA and peptides containing Lys and Arg sidechains, and led to the formulation of the electrostatic-hydrogen bond switch model [18]. Additionally, mean field calculations led to further support for this model [24,25]. While numerous PA binding proteins are known [6,26,27], no specific PA binding domain has been identified [28]. This is in contrast to anionic lipids such as the PIPs, for which numerous lipid binding domains are known [29], and which allow for bioinformatic studies to identify novel lipid binding proteins [30]. In the case of PA, the specific binding of proteins is likely driven by the unique physical chemical properties of this lipid, namely the ionization properties and spontaneous curvature. In the case of DGPP, only cytosolic GAPDH from *Hordeum vulgare* has been identified as a potential binding protein [31]. The Lys residues in the NAD+ binding site of GAPDH have been reported to be very active in numerous activities including the stable bonds formed with the phosphate group of phosphatidylserine (PS) [32], and may be responsible for the identification of GAPDH in the DGPP binding screen. The implication of GAPDH-DGPP binding has not been further explored. Further work on the identification of unique DGPP binding proteins is thus an important area of future research. 

Calcium (Ca^2+^) and magnesium (Mg^2+^) are divalent cations with prominent intracellular functions [33,34]. Calcium’s role as a secondary messenger is well recognized [35], and Mg^2+^ is the most abundant divalent cation in living cells with a vital role in plant signaling [36]. One of their mechanistic pathways is via interaction with zwitterionic and anionic lipids in the cell to mediate important physiological activities [37,38]. Changes in cytosolic calcium concentrations have been reported in stress responses [39,40] that can affect production of minor lipids in the membrane. Additionally, changes in lipid composition can alter calcium concentration [41]. Calcium can bind simultaneously to multiple anionic binding sites to affect lipid density and packing, change the conformation, and dehydrate the lipid headgroup region of the affected membrane [42,43]. The interaction of calcium and magnesium with membrane lipids is determined, in part, by the difference in their hydration energy [44]. The calcium ion more easily releases its associated water molecules and can therefore interact more strongly with anionic membrane lipids [44]. Magnesium is known to promote the fusion of lipid vesicles, regulate ion channels and signaling pathways [45,46]. 

The ionization properties of PI(4,5)P_2_ were differentially affected by the presence of these two divalent cations [47]. Compared to magnesium, calcium had a stronger interaction with the more accessible 5-phosphate of PI(4,5)P_2_ leading to clustering (domain formation) of this critical membrane lipid [44]. In comparison, magnesium induced clustering of PI(4,5)P_2_ only occurs at much higher concentrations [44]. As seen in PI(4,5)P_2_, phosphate position and orientation together with the inherent properties of these divalent cations affect their binding. Margutti et al. showed that calcium affected the surface pressure of DGPP (containing) monolayers differentially at pH 5 and 8 using Langmuir monolayers [19]. No literature exists on the interaction of Mg^2+^ with DGPP and how this compares with Ca^2+^.

DGPP is expected to interact with positively charged intracellular molecules to exert its function. To gain insight into these interactions, we use solid state ^31^P NMR to examine the impact of KALP23 and WALP23, transmembrane α-helical peptides [48,49,50] that differ in the presence of flanking lysine and tryptophan residues respectively, on the charge of DGPP. In addition, the effect of the important divalent Ca^2+^ and Mg^2+^ cations on the charge of DGPP together with the cationic lipid dioleoyl trimethylammonium-propane (DOTAP) and ethyl-phosphatidylcholine (EtPC) were also investigated. Here we find that KALP23 altered the charge of DGPP significantly as compared to WALP23. Surprisingly, the effect of DOTAP and EtPC on the charge of DGPP were very small. This observation can be rationalized by the deprotonation of the inner phosphate of the pyrophosphate of DGPP. While Ca^2+^ has a significant effect on the ionization of DGPP, we do not observe a differential interaction when comparing Ca^2+^ and Mg^2+^ ions, opposite to what we previously observed for PI(4,5)P_2_ [44,47].

## 2. Materials and Methods

### 2.1. Materials

1,2-dioleoyl-sn-glycero-3-phosphatidylcholine (DOPC), 1,2-dioleoyl-3-trimethylammonium-propane (DOTAP), 1,2-dioleoyl-sn-glycero-3-ethylphosphocholine (EtPC), and 1,2-dioleoyl-sn-glycero-3-pyrophosphate (DGPP) were purchased from Avanti Polar Lipids (Birmingham, AL, USA). Lipids were dissolved in a 2:1 mixture of chloroform and methanol. Lipid stocks were prepared gravimetrically and their concentrations were confirmed by phosphate assay (according to Rouser [51]). Thin layer chromatography (TLC) was used to confirm the purity of prepared lipid stocks and only stocks that showed a single spot on the TLC plate were used. CaCl_2_, MgCl_2_, and A23187 ionophore were purchased in powder form from Sigma Aldrich (St Louis, MO, USA). A23187 ionophore was dissolved in pure ethanol. KALP23 and WALP23 peptides were a generous gift from Antoinette Killian. HPLC-grade water was purchased from Fisher Scientific.

### 2.2. Sample Preparation

NMR samples were prepared as described in Graber and Kooijman [52]. Dry lipid films of 10–15 μmol were prepared by mixing appropriate volumes of lipid stock solution (in 2:1 chloroform:methanol) in borosilicate glass tubes. For samples containing KALP23 or WALP23, the peptides were dissolved in 2,2,2-Trifluoroethanol (TFE) and added to the mixed lipid solution at a 100:2 or 100:4 lipid to peptide ratio. TFE is used here to maintain peptide secondary structure in our organic solvent mixtures. Chloroform, methanol, and TFE (where appropriate), in lipid or lipid–peptide mixtures were evaporated under nitrogen and the resulting lipid or lipid–peptide samples were left in the borosilicate glass and further dried overnight in a high vacuum at 45 deg Celsius to remove residual traces of organic solvent. Lipid or lipid–peptide films were hydrated with 2 mL of buffer and vortexed to form multilamellar vesicle dispersions. The following buffers were used for the indicated pH ranges; 20 mM citric acid, 30 mM MES for pH 4–6.5, 50 mM HEPES for pH 6.5–8.5, and 50 mM glycine for pH 8.5–10. Buffers also contained 100 mM NaCl and 2 mM EDTA to complex divalent cations, except divalent cation containing buffers which lacked EDTA. Experiments performed at a constant pH value of 7.20 ± 0.05 used a more concentrated 100 mM HEPES buffer to minimize pH variation. For samples that contained divalent cations, the cations were added to the buffer from concentrated aqueous stock solutions. In addition, A23187 ionophore was added at a 1:1000 ionophore-to-lipid ratio. Lipid dispersions were exposed to two freeze–thaw cycles to remove metastable states. The freeze–thaw procedure consisted of quickly freezing the lipid suspension in a mixture of ethanol and dry ice, and then thawing in warm water while occasionally vortexing the sample. The pH of each NMR sample was carefully measured using a Sentron Intelli pH probe and standard pH meter, and this measured bulk pH was used to prepare the pH titration curves. Lipid samples were spun down at 15,000 rpm at 4 °C for 45–60 min to create a concentrated lipid pellet. The lipid pellets were loaded into 4 mm zirconium MAS NMR sample tubes for NMR spectroscopy.

### 2.3. NMR Spectroscopy

^31^P NMR spectra were recorded as previously described [52]. An 85% H_3_PO_4_ standard solution was run before each sample solution to act as an external reference. Sample spectra were recorded under stable spinning conditions (5 kHz) at 21 ± 1.0 °C. Generally, 15,000 to 50,000 scans were collected. We only determined the upper part (pK_a2_) of the full titration curve for the systems explored here. We chose this to save precious peptide and lipid, and we previously showed that determining only the ionization behavior in the physiological pH range does not significantly affect the pK_a2_ value [53]. After the MAS spectra was recorded, the phase behavior of the lipid sample was determined via static experiment for select samples. The static experiments were recorded on the same probe and aided by low-power proton decoupling (the spinal 64 pulse program was used for proton decoupling). For each static experiment, 20,000 to 150,000 scans were collected.

### 2.4. Charge Determination from Titration Curve Data

The charge of the phosphomonoester of DGPP was determined by assuming that the chemical shift value is a weighted average of the chemical shifts of the singly and completely deprotonated state of the respective phosphate group [54]. The chemical shift can then be expressed as:

(1) $$\delta_{i}^{obs}=f_{i,p\;}\delta_{i,p}+\left(1-f_{i,p}\;\right)\delta_{i,d}$$

where $\delta_{i}^{obs}$ is the pH dependent chemical shift of the phosphomonoester group *i*, while *δ_i,p_* and *δ_i,d_* are chemical shifts of the singly protonated and completely deprotonated phosphate group. Equation (1) can be re-arranged to

(2) $$f_{i,p}=\frac{\delta_{i}^{obs}-\delta_{i,d}}{\delta_{i,p\;}-\delta_{i,d}\;}$$

where *f_i,p_* is the degree of protonation of the phosphate group *i*. The pK_a2_ value for the phosphomonoester of DGPP phosphate was determined based on a Henderson–Hasselbalch derived fit, as in Kooijman et al. [53], using the relation:

(3) $$\delta =\frac{\delta_{i,p}10^{pK_{a}-pH}+\delta_{i,d}}{1+10^{pK_{a}-pH}}$$

### 2.5. Statistical Analysis

Experiments for DGPP’s interaction with KALP23, WALP23 and the control were performed at least three times. Those for DGPP’s interaction with divalent cations together with its control were performed at least 7 times. These were then used to calculate the means, standard deviations (SD), and standard errors of means (SEM). SigmaPlot (Systat Software Inc, San Jose, CA, USA) was used to plot the graphs using the means and SD. We performed one-way analysis of variance (ANOVA) followed by Dunnett’s test. *p* values < 0.001 were considered statistically significant.

## 3. Results and Discussion

**Figure 1:**
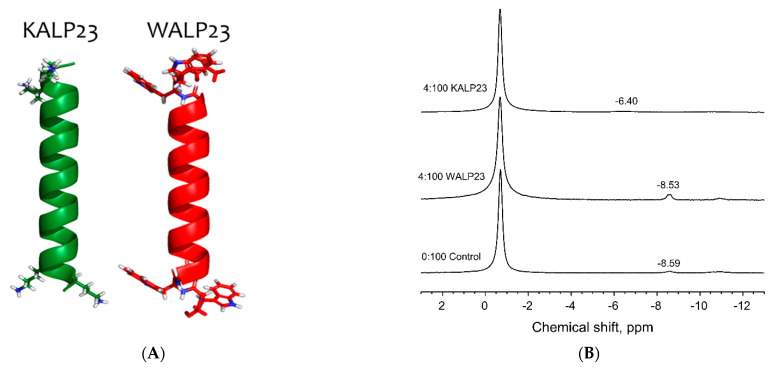

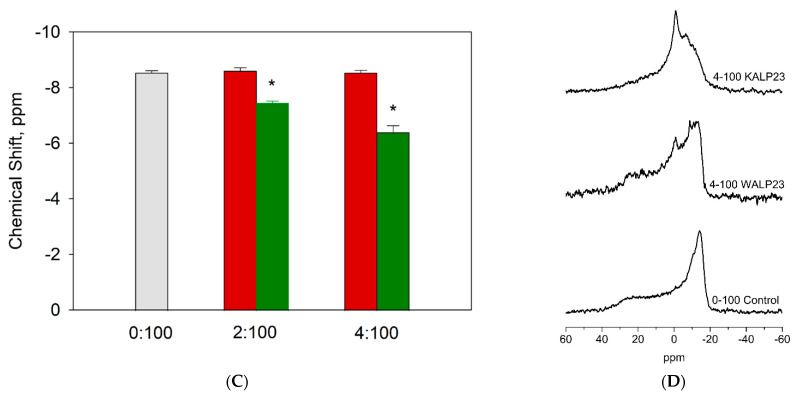
Effect of transmembrane peptides on the chemical shift of DGPP. (**A**) Ribbon models for the transmembrane Alanine and Leucine (AL) peptides KALP23 and WALP23 with flanking lysine and tryptophan residues. (**B**) Representative solid-state ^31^P NMR spectra for control (DOPC:DGPP (95 mol%:5 mol%)), WALP23 and KALP23 at a 4:100 peptide–lipid ratio. (**C**) Quantified changes in the chemical shift of the phosphomonoester of DGPP in the presence of KALP23 and WALP23, compared to control, as a function of peptide–lipid ratio. Error bars show the standard deviation determined from at least three unique samples (* *p* < 0.001). (**D**) Representative static ^31^P NMR spectra for NMR samples from B that show samples formed lipid bilayers. Chemical shift values are relative to an 85% H3PO4 external reference.

The pyrophosphate headgroup of DGPP is unusual in membrane phospholipids, and we previously showed that in model membranes, only the phosphomonoester of DGPP carries charge, while the inner phosphate remains largely protonated [17]. We also showed that the phosphomonoester of DGPP follows the electrostatic hydrogen bond switch model, as PE increases the charge (decreases the pK_a_). However, how the pyrophosphate headgroup of DGPP interacts with cationic moieties in proteins or with divalent cations has only received limited attention. Here we investigate the ionization of DGPP in the presence of transmembrane peptides that are anchored in the membrane by specific flanking residues (see Figure 1A). Additionally, we study the effect of two crucial divalent cations on the charge (i.e., chemical shift) of DGPP.

### 3.1. Lysine Residues Interact Strongly with the Pyrophosphate Headgroup of DGPP and Increase Its Charge

Many peripheral membrane proteins (and some integral proteins as well) rely on cationic domains containing lysine and/or arginine residues to interact with anionic phospholipids. These interactions are often considered to be electrostatic in nature, but we have extensively shown that hydrogen bond formation is also an important driver of membrane recruitment and localization, especially in the case of PA [18,24]. 

The electrostatic-hydrogen bond switch model sums up these interactions for PA (and any phosphomonoester group for that matter) [18]. The anionic membrane potential recruits cationic proteins to the membrane. Depending on the potential, and other interactions (hydrogen bonding, hydrophobic, etc.), the protein then either binds or samples a different patch of the membrane. When a PA-binding protein binds to PA, it is able to hydrogen bond to the phosphomonoester headgroup. This leads to an increase in the negative charge of PA from −1 to −2, further strengthening the electrostatic interaction and thus docking of the protein onto the membrane. Both PA and DGPP contain a phosphomonoester in their headgroup, and DGPP may therefore use a similar mechanism to interact with membrane proteins.

To investigate this, we used the transmembrane peptide KALP23 as a model for cationic protein domain interaction with DGPP. The membrane spanning α-helix of KALP23 [48,49,50] consists of a repeating alanine-leucine motif and is flanked on either side by two lysine residues (see Figure 1A). The hydrophobic alanine-leucine repeat and positively charged flanking residues ensure the transmembrane orientation of the peptide and position the lysine residues in the right location to interact with the pyrophosphate of DGPP [48,55]. As a control, we used WALP23, which is flanked by two tryptophan residues on either side of the peptide (see Figure 1A). While the tryptophan residues anchor the peptide in a transmembrane orientation [48], it is not charged, and hence WALP23 cannot interact with DGPP through electrostatic interactions. The chemical shift (CS) of the DGPP phosphates was monitored for two concentrations of peptide (see Figure 1B,C). There is no significant change in CS for the mixtures containing WALP23, indicating that the presence of the peptide in the membrane does not affect the ionization of DGPP. When KALP23 is added to the membrane, we observe a concentration dependent increase (to more downfield values) for the CS of the DGPP phosphomonoester, indicating increased deprotonation. Additionally, we observe a significant broadening of the phosphomonoester peak of DGPP. 

**Figure 2:**
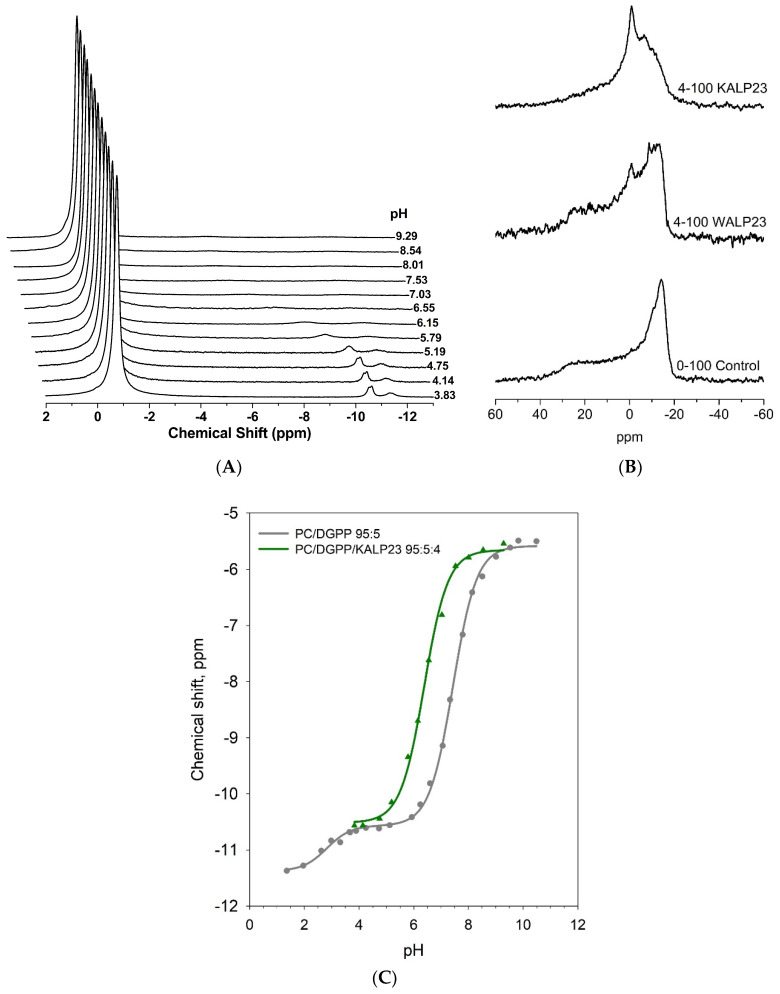
Ionization curve for DGPP in the presence of KALP23. (**A**) ^31^P MAS NMR spectra as a function of pH for 5 mol% DGPP in 95% DOPC vesicles. (**B**) Static ^31^P NMR spectra for low, medium, and high pH values recorded for samples from A. (**C**) Ionization curve for the phosphomonoester of DGPP as a function of pH. The titration curve for DGPP in the presence of KALP23 (green) is compared against the control curve in the absence of KALP23 (grey), taken from [17]. The solid lines are from a Henderson–Hasselbalch fit of the ionization data (see Methods).

To further investigate this effect, the ionization of DGPP in the presence of KALP23 was observed over the pH range from 4 to 10 (see Figure 2A). The peaks were assigned as in our previous work, with the PC peak furthest downfield followed by the phosphomonoester peak and finally the phosphodiester peak. Figure 2B shows static ^31^P NMR spectra for selected pH values that show our model systems form lipid bilayers, characterized by a high field peak, and low field shoulder, under our experimental conditions (also see Figure 1D). As the pH increases the DGPP phosphomonoester peak shifts downfield as the phosphomonoester is deprotonated. As observed in the constant pH measurements, we observe that around pH 6.1–6.5, the phosphomonoester peak significantly broadens but does not disappear completely, allowing for a pH titration curve to be constructed. This significant broadening of the phosphomonoester peak of DGPP in the presence of KALP23 (Figure 2A) is not observed in the case of PA. The largest contribution to NMR peak line width is from T1 and T2 relaxation, which are strongly dependent on the dynamics of the nuclei [56,57,58]. In this case, the increased linewidth suggests that the lysine residues of KALP23 bind tightly to the pyrophosphate of DGPP resulting in altered relaxation dynamics. This tight binding is not observed in the case of PA as indicated by a lack of line broadening, and is likely to have important implications for the interaction of DGPP with peripheral membrane proteins.

This observation suggests that PA may have weaker electrostatic and hydrogen bond interactions with membrane proteins compared to DGPP (see further discussion below).

By plotting the CS values from Figure 2A with respect to pH, we obtain an ionization curve. This ionization curve is compared against our previous data for 5 mol% of DGPP in 95 mol% DOPC (Figure 2C). In the presence of KALP23 there is a significant shift of the ionization curve to the left, indicating a lower pK_a2_ value and hence higher DGPP charge (at constant pH). Based on a non-linear fit using Equation (3) (derived from the Henderson–Hasselbalch equation and the assumption that the observed chemical shift values are weighted averages of the chemical shifts of the singly dissociated and doubly dissociated states) we determine a pK_a2_ value of 6.38 ± 0.04 (see Table 1). This is a shift of 1.06 ± 0.06 pH values (logarithmic scale) with respect to the mixture in the absence of KALP23 (pK_a_ 7.44 ± 0.02) [17]. This shift is larger compared to what we observed for PA in the presence of KALP23 (0.78 ± 0.09 pH values) [18], and suggests that DGPP interacts with KALP23 through a similar electrostatic-hydrogen bond switch mechanism. Interestingly, the interaction between the primary amine in the lysine residues of KALP23 is significantly “stronger” than those of PE. For a more than 2-fold larger concentration of PE compared to the lysines in KALP23, we find a pK_a2_ shift of only 0.73 ± 0.04 [17] (similar to what we observe for the PA-KALP23 interaction), compared to 1.06 ± 0.06 for KALP23. This observation shows that both hydrogen bond formation, and overall cationic charge (PE is zwitterionic) are important for this interaction, and that lysine residues interact “more strongly” with a pyrophosphate (as in DGPP) compared to a phosphomonoester (as in PA).

### 3.2. Positive Charge Alone Does Not Significantly Affect the Charge of the Phosphomonoester of DGPP

In the previous section we looked at the interaction of DGPP with the cationic peptide KALP23. While the increased deprotonation of DGPP in the presence of KALP23 suggests the formation of a hydrogen bond, it is also possible that KALP23 exerts its effect on DGPP ionization simply via its effect on the membrane potential. The cationic charge contributed by the lysine residues reduces the negative membrane potential and is likely to have a significant impact on ionizable groups at the membrane. The negative surface potential (due to the presence of DGPP) of the membrane lowers the pH at the membrane interface compared to the bulk pH. The presence of positively charged proteins or peptides at the membrane will decrease this negative membrane surface potential. To examine the effect of altering the membrane potential, the ionization of DGPP was examined in the presence of the cationic lipids DOTAP and EtPC. 

**Figure 3:**
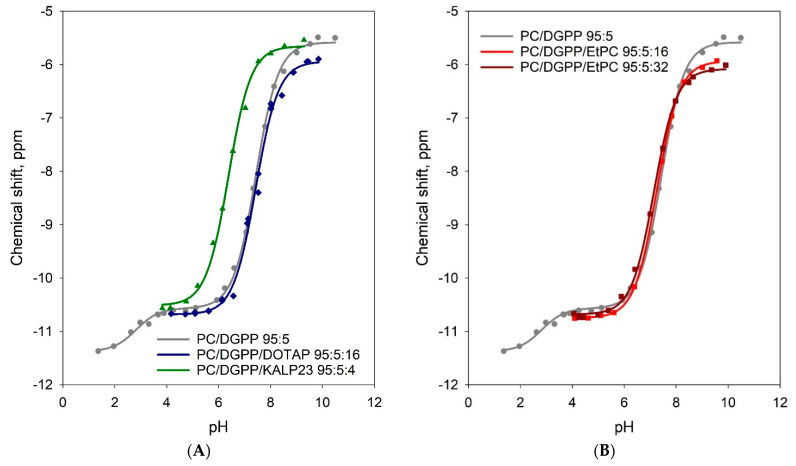
Solid-state MAS ^31^P NMR-based titration curves for DGPP in the presence of cationic lipids. (**A**) DOTAP, blue diamonds and lines. Also shown are the KALP23 data from Figure 2. (**B**) EtPC 16 and EtPC 32 are shown in light red and dark red squares and lines, respectively. The DOTAP data in Figure 3A and Figure S4A are compared with our previous results for KALP23 and the 95:5 DOPC/DGPP control. Surprisingly, we observe no effect on the ionization behavior and charge of the phosphomonoester of DGPP due to the presence of the cationic DOTAP (the titration and especially the protonation curve for DOTAP completely overlaps with that of the PC/DGPP control). This is opposite to what we previously observed for PA. In the presence of DOTAP the ionization curve for PA moved to lower pH values, albeit less than in the presence of KALP23, indicating a lower pK_a2_, and hence a higher negative charge for the phosphomonoester as expected based on simple electrostatics. The effect of positive charge in the quaternary amine of DOTAP accounted for ~40% of the change in charge of PA [18] compared to the change in charge induced by KALP23 (due to both positive charge and hydrogen bonding). Since DOTAP has no effect on the ionization of the phosphomonoester of DGPP, something novel must be happening at the lipid headgroup–aqueous interface. To investigate this further, we used a cationic lipid that has a positive charge that is located further away from the hydrophobic interior of the membrane. In DOTAP, the positive charge is located in the quaternary amine, which is likely close to the pyrophosphate of DGPP. We thus chose a cationic PC where the phosphate is conjugated with an ethyl group, hence ethyl-PC (EtPC). The quaternary amine of this EtPC is located further away from the hydrophobic interior of the membrane and closer to the aqueous interface.

Figure 3A shows the titration curve for the phosphomonoester of DGPP in a 95:5:16 mixture of PC:DGPP:DOTAP compared to same mixture without DOTAP (previous data, [17]). Since KALP23 contains four positive charges per peptide, while DOTAP has only one, we used a lipid-to-cation (DOTAP) ratio of 100:16 to be able to directly compare the KALP23 and cationic lipid data. The full ^31^P NMR titration curves are shown in supplementary data Figure S1. To consistently evaluate the significance of changes in pK_a_, we also calculate the degree of protonation and the charge at pH 7.2 for each of our titration data, see Figure S4, and Table 1.

Figure 3B shows the titration curves for the phosphomonoester of DGPP in a 95:5:16 and 95:5:32 mixture of PC:DGPP:EtPC. Again, the ^31^P NMR spectra as a function of pH are shown in the supplementary data, Figures S2 and S3. The protonation curves are shown in Figure S4. Interestingly, we now observe a small effect on the pK_a2_ and charge of the phosphomonoester of DGPP. For the 95:5:16 mixture the pK_a2_ is reduced to 7.25 compared to 7.44 for DGPP in a pure PC matrix, and doubling the amount of EtPC results in a further shift of the pK_a2_ to 7.14 (see Table 1). The effect on charge of the phosphomonoester is not significant for the 95:5:16 lipid mixture but becomes significant (difference of 0.15) for the mixture containing twice as much EtPC. The overall impact on the pK_a2_ of DGPP is thus surprisingly small compared to the large impact observed in the presence of KALP23 (where the pK_a_ decreased by 1.06 pH units).

### 3.3. Proposed Model for the Effect of Positive Charge on the Ionization of DGPP

**Figure 4:**
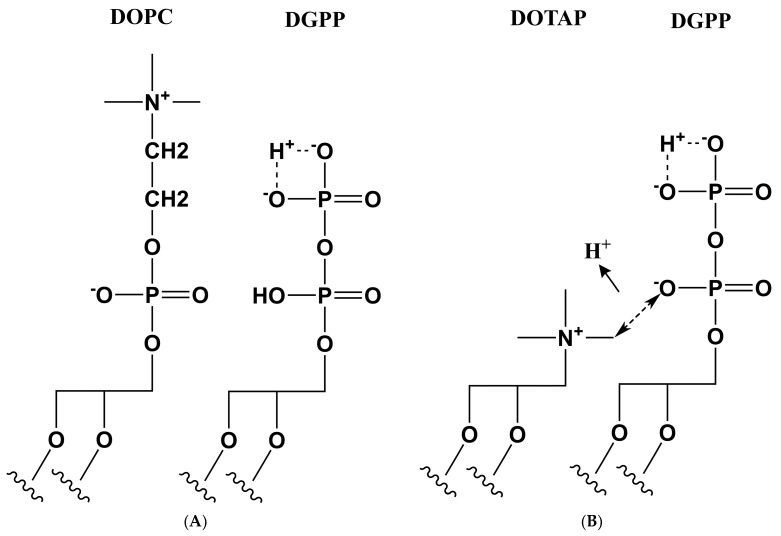
(**A**) Schematic showing DOPC and DGPP in the membrane with no direct electrostatic interaction between DGPP’s inner phosphate and the phosphate of DOPC. (**B**) Schematic showing the electrostatic interaction between the positively charged amine of DOTAP and the inner phosphate of DGPP leading to its ionization (removal of the proton).

How do we reconcile the observed ionization behavior of the pyrophosphate of DGPP with basic electrostatics? Physics tells us that addition of positive charge to a surface should impact the surface potential and thus the ionization behavior of ionizable groups at this interface. Exactly as we have previously observed for PA [18]. Here, no such effect on the ionization of the DGPP phosphomonoester is observed. The most plausible explanation for this unusual behavior is the following: In model membranes containing only zwitterionic and anionic membrane lipids, the inner phosphate of the pyrophosphate headgroup remains protonated, as discussed previously [17]. Addition of a positive charge, right next to this pyrophosphate due to DOTAP and EtPC, now removes this proton from the inner phosphate resulting in an additional negative charge of DGPP. However, this negative charge is balanced by the positive charge on the DOTAP or EtPC molecule, resulting in no, or only very small local changes in the electrostatic membrane potential. A cartoon of this model is shown in Figure 4. This also explains the strong interaction of the lysine residues in KALP23 with the pyrophosphate headgroup. Not only is the charge in the phosphomonoester increased (as in the case of PA), but the inner phosphate now also becomes deprotonated, leading to a further increase in the total charge of DGPP (see Table 1). The almost −3 charge of DGPP (coupled to the hydrogen bonding with lysine residues) compared to the charge of −2 for PA also explains why we observe partial immobilization of the pyrophosphate headgroup as evidenced by the line broadening (see Figure 2).

### 3.4. Implications for DGPP—Protein Interactions

Our results have important implications for how DGPP may interact with membrane proteins and other membrane lipids. In the following discussion we will focus primarily on protein–lipid interactions for DGPP. Our observation of ^31^P NMR line width broadening coupled with the large shift in the titration curve due to KALP23 suggests that lysine residues have a much stronger interaction with the pyrophosphate of DGPP than the single phosphomonoester of PA. The primary amine of lysine likely deprotonates the entire pyrophosphate resulting in a total charge of DGPP of −3 (see Table 1), unlike the total charge for PA of −2. Couple this much larger charge with hydrogen bond formation (as summarized in the electrostatic-hydrogen bond switch model) and you get a much stronger binding interaction (higher binding constant) for lysine and presumably arginine residues with DGPP than PA [18]. It is interesting to note that PA binding proteins do not bind more strongly to DGPP as a result of the increased interaction between cationic and hydrogen bond forming amino acids and the pyrophosphate headgroup. We showed this previously for a random selection of PA binding proteins where we found either similar or significantly less binding to DGPP compared to PA [60]. This makes sense when we consider another critical lipid physical chemical property, namely spontaneous curvature. We have extensively shown that PA binding proteins require negative membrane curvature (or in other words lipids with negative spontaneous curvature such as PE or PA itself) to efficiently bind to a lipid membrane. DGPP, however, is a lipid with essentially zero spontaneous curvature [17], and hence PA binding proteins, while having a stronger electrostatic and hydrogen bond interaction with its headgroup, do not bind more efficiently to DGPP over PA. In the case of PA signaling in plants or parasites this means that conversion of PA, formed due to a biotic or abiotic signal, to DGPP may result in an attenuation of the PA signal. Fine tuning the amount of PA and DGPP in a membrane could thus be a sensitive regulator for protein membrane localization. One important avenue for future research is to carefully investigate whether there are indeed specific DGPP binding proteins, and then to explore in detail the interaction of those proteins with both PA and DGPP. Only one published study has looked at DGPP binding proteins to date [31].

### 3.5. The interaction of DGPP with Divalent Cations

Divalent cations have the potential to interact with anionic phospholipids and alter phospholipid ionization due to changes in membrane potential. Previous work with the anionic phospholipid PI(4,5)P_2_ has shown that calcium interacts strongly with the phosphomonoesters in its headgroup (specifically the 5-phosphate), while magnesium interacts much more weakly [47]. The significant difference in affinity was suggested to be due to differences in hydration energy and ion size [44].

**Figure 5:**
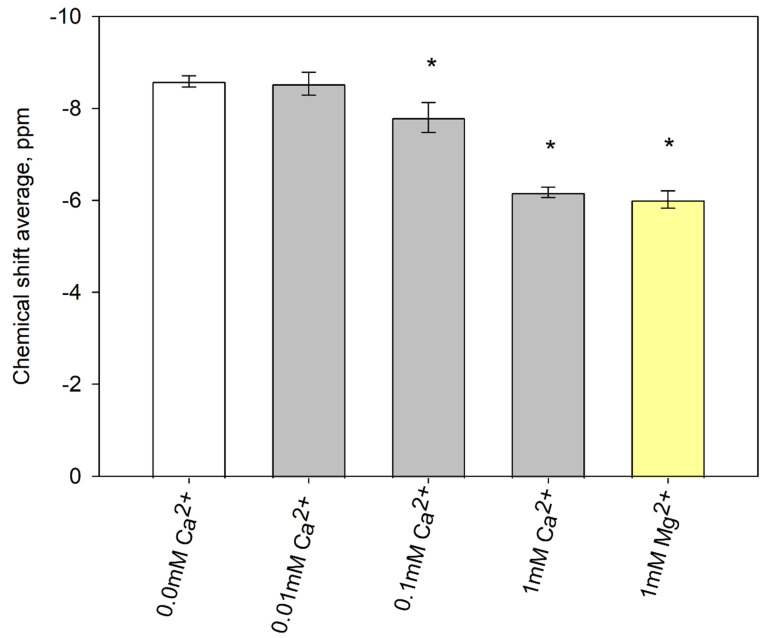
DGPP Ionization in the presence of Divalent Cations. The DGPP phosphomonoester peak chemical shift value is plotted for varying cation concentrations. Chemical shift values are obtained from ^31^P MAS NMR spectra of DOPC:DGPP (95%/5%) MLVs in buffer at pH 7.2 ± 0.1 with 100 mM NaCl. Samples contained 0–1 mM Ca^2+^ or 1 mM Mg^2+^. Error bars show the standard deviation measured from at least seven samples. * *p* < 0.001.

Here we investigated the ionization of DGPP in the presence of varying concentrations of calcium and magnesium ions. The ^31^P chemical shift of the DGPP phosphomonoester was determined in DOPC/DGPP (95%/5%) MLVs at pH 7.2 ± 0.1 and 100 mM NaCl (see Figure 5). As the calcium concentration increases, the chemical shift for the DGPP phosphomonoester increases, indicating increased deprotonation. For calcium levels below 0.1 mM there is no significant effect, but for 1 mM calcium there is a change of more than 2 ppm, suggesting almost complete deprotonation of the phosphomonoester group. The relative ionization of DGPP can be estimated based on the change in chemical shift. The estimated DGPP charge is shown in Table 2. Please note that this charge assumes that the presence of Ca^2+^ or Mg^2+^ does not by itself impact the DGPP chemical shift (this assumption proved valid for PIP_2_ [47]). The DGPP phosphodiester is unaffected by the calcium content. Interestingly, with 1 mM Mg^2+^ there is as large an effect as for 1 mM Ca^2+^. This suggests that Ca^2+^ and Mg^2+^ bind to DGPP with equally high affinity. This is notably different from cation binding to PIP_2_, where a strong preference was shown for Ca^2+^ and indicates that the DGPP binding site is unique compared to PIP_2_ and can accept both ions without preference [44].

## 4. Conclusions

In this work, we have characterized the interaction between DGPP and several cationic membrane components. We found that the charge of DGPP is affected by the cellular cations Ca^2+^ and Mg^2+^, as expected. Unlike for PI(4,5)P_2_, however, DGPP is not specifically sensitive to Ca^2+^, but interacts with Ca^2+^ or Mg^2+^ to a similar degree. At least as far as our charge analysis can differentiate. These data also do not show a specific binding site for Ca^2+^, but additional experiments (e.g., X-ray fluorescence from Langmuir monolayers) [61] are needed to fully answer this question.

Based upon our investigation of the DGPP-KALP23 interaction, we suggest that DGPP interacts with membrane proteins in a similar manner to PA, i.e., via the electrostatic-hydrogen bond switch model. However, the interaction of Lys residues with DGPP is significantly stronger than that with PA. This may suggest that PA and DGPP compete for the same protein binding sites. However, PA binding proteins do not bind better to DGPP as we have shown and hence DGPP formation is likely to result in an attenuation of PA signaling. An unanswered question is how PA and DGPP interact when present in the same bilayer leaflet. Monolayer work suggests that PA and DGPP may form domains [19] but this needs to be corroborated in model lipid bilayers. It is tempting to speculate however that fine tuning of the concentration of PA and DGPP in a given membrane may thus be one mechanism by which to sensitively control PA—Protein interactions and lipid signaling. Taken together, our results give us important insights into how DGPP interacts with critical cellular components.

## Figures and Tables

**Table 1:** pK_a2_ values and charges for DGPP in DOPC, DOPC/KALP23, DOPC/DOTAP, DOPC/EtPC bilayers ^a^.

Lipid Composition	pK_a2_^a^	Phosphomonoester Charge at pH 7.2	Total Charge at pH 7.2
		(±0.1) ^b^	(±0.1) ^b^
DOPC/DGPP 95:5	7.44 ± 0.02	−1.38	−1.38
DOPC/DGPP/KALP23 95:5:4	6.38 ± 0.04	−1.84	−2.84
DOPC/DGPP/DOTAP 95:5:16	7.43 ± 0.03	−1.37	−2.37
DOPC/DGPP/EtPC 95:5:16	7.25 ± 0.01	−1.47	−2.47
DOPC/DGPP/EtPC 95:5:32	7.14 ± 0.02	−1.53	−2.53

^a^ The error indicated for the pK_a2_ values comes directly from the nonlinear curve fit (and is a lower limit). ^b^ The error in the total charge is an upper estimate which follows from the reproducibility in the calculated degree of protonation *f* taken from [59]. This error in the total charge is a conservative estimate, i.e., the actual error is likely to be lower.

**Table 2:** Calculated charge for DGPP in the presence of divalent cations. Estimated charge values for the DGPP phosphomonoester in the presence of varying amounts of divalent cations at pH 7.20 ± 0.05 and 100 mM NaCl. Charge values are calculated using Equation (2) and assuming that the chemical shift of the protonated and deprotonated states of the phosphomonoester are not influenced by the addition of divalent cations.

Cation Content	Phosphomonoester Charge ± 0.1 at pH 7.20
0 mM	−1.40
0.01 mM Ca^2+^	−1.41
0.1 mM Ca^2+^	−1.55
1 mM Ca^2+^	−1.88
1 mM Mg^2+^	−1.91

## Data Availability

The data presented in this study are available in the article and its Supplementary Data.

## Supplementary Materials

The following supporting information can be downloaded at: https://www.mdpi.com/article/10.3390/cells11020290/s1, Figure S1: ^31^P MAS NMR spectra as a function of pH for 5 mol% DGPP in DOPC/DOTAP (95:16) vesicles.; Figure S2: ^31^P MAS NMR spectra as a function of pH for 5 mol% DGPP in DOPC/EtPC (95:16) vesicles; Figure S3: ^31^P MAS NMR spectra as a function of pH for 5 mol% DGPP in DOPC/EtPC (95:32) vesicles; Figure S4: (A) Degree of protonation (fp) as a function of pH for DGPP in the presence of KALP23 (green) and DOTAP (blue) is compared for 5mol % DGPP in 95%DOPC vesicles in grey. (B) Degree of protonation (fp) as a function of pH for DGPP in the presence EtPC 16 (light red) and EtPC 32 (deep red) is compared for 5 mol% DGPP in 95%DOPC vesicles in grey.
