# Supplementary material for: The Electrostatic Basis of Diacylglycerol Pyrophosphate—Protein Interaction
Source: Cells. 2022 Jan 15;11(2):290. doi: 10.3390/cells11020290 (PMC8774204; doi:10.3390/cells11020290)
Supplement: Supplementary file 1 [file cells-11-00290-s001.zip › cells-1486477-supplementary.pdf]

SUPPLEMETARY DATA

The Electrostatic Basis of Diacylglycerol Pyrophosphate – Protein Interaction.

Zachary Graber[1,2*], Desmond Owusu Kwarteng[3*], Shannon M. Lange[3], Yannis Koukanas[3], Hady Khalifa[3], Jean W. Mutambuze[3] and Edgar E. Kooijman[3*]

[1]Department of Chemistry & Biochemistry, Kent State University, 800 E. Summit St., Kent, OH 44242, USA.
[2]School of Natural and Social Sciences, Mount Vernon Nazarene University, 800 Martinsburg Road, Mount Vernon, OH 43050, USA
[3]Department of Biological Sciences, Kent State University, 800 E. Summit St., Kent, OH 44242, USA;

[*]Correspondence: Zachary.Graber@mvnu.edu, dowusukw@kent.edu, ekooijma@kent.edu

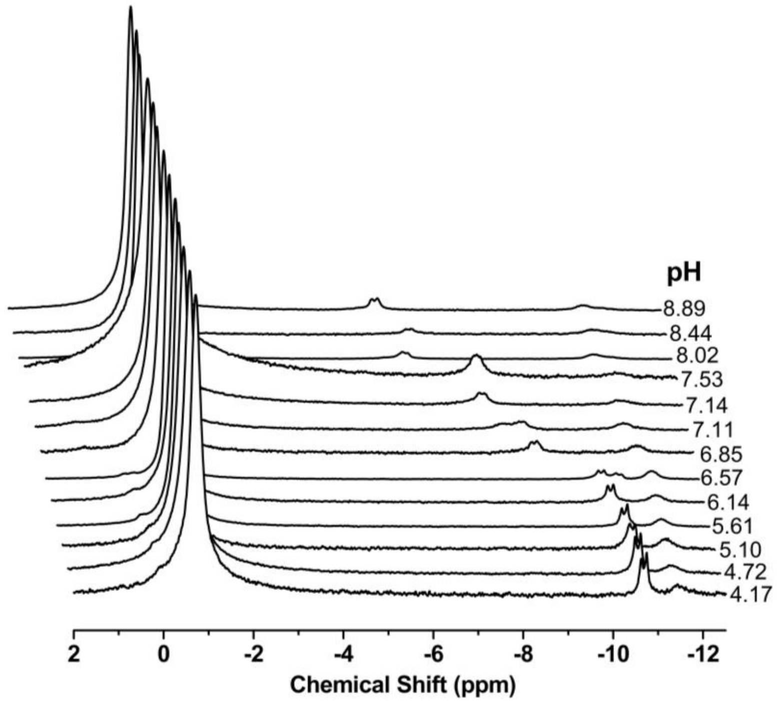

Figure S1: ^{31}P MAS NMR spectra as a function of pH for 5 mol% DGPP in DOPC/DOTAP (95:16) vesicles.

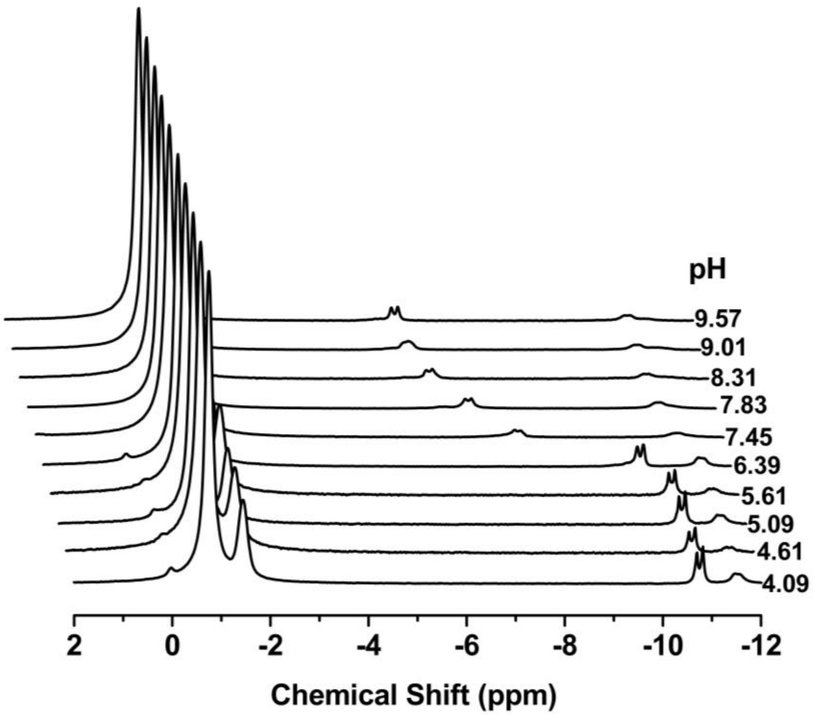

Figure S2: ^{31}P MAS NMR spectra as a function of pH for 5 mol% DGPP in DOPC/EtPC (95:16) vesicles.

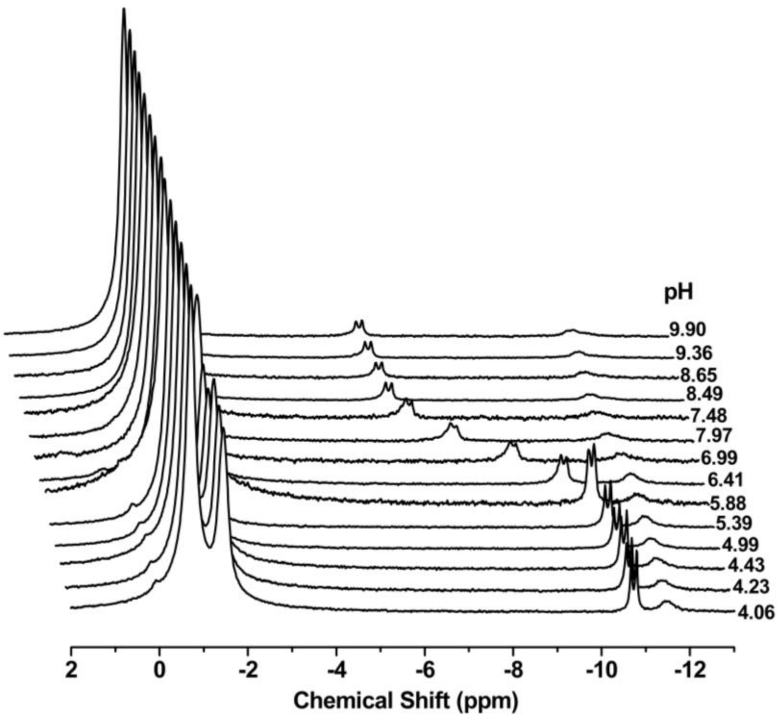

Figure S3: ^{31}P MAS NMR spectra as a function of pH for 5 mol% DGPP in DOPC/EtPC (95:32) vesicles.

Note that in Figures S1 through S3 we observe a small amount of lipid breakdown as shown by the small peak to the left of the large DOPC (bilayer matrix) peak. This peak coincides with that of PA as it titrates with the change in pH and most likely represents DGPP breakdown during the course of our NMR experiments. Based on peak area we estimate this breakdown to represent less than 1 mol% of all lipids in the sample and less than 15 mol% of the DGPP in each sample. This breakdown was not observed in the KALP23 or WALP23 experiments and its exact cause is unclear. The overall conclusions of this work are not significantly affected by this breakdown.

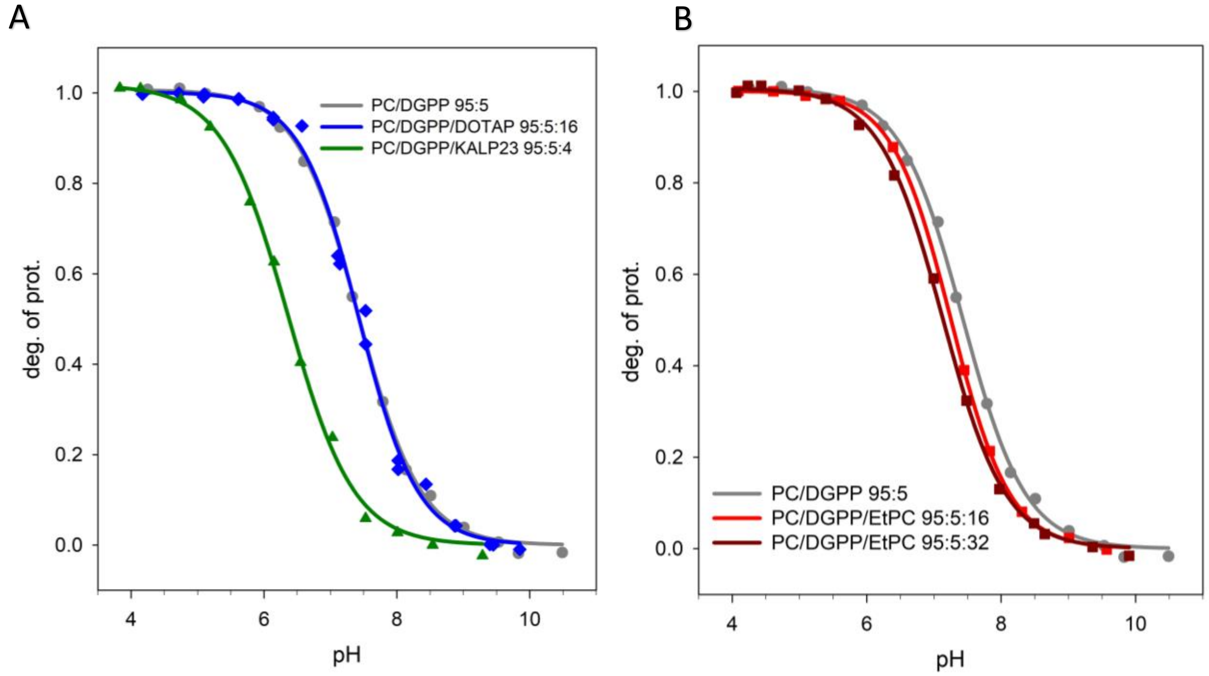

Figure S4A: (A) Degree of protonation (f_p) as a function of pH for DGPP in the presence of KALP23 (green) and DOTAP (blue) is compared for 5mol % DGPP in 95%DOPC vesicles in grey. (B) Degree of protonation (f_p) as a function of pH for DGPP in the presence EtPC 16 (light red) and EtPC 32 (deep red) is compared for 5mol % DGPP in 95%DOPC vesicles in grey.
